# Safety and efficacy of the second-generation cryoballoon for left atrial appendage electrical isolation in canines

**DOI:** 10.1093/europace/euae100

**Published:** 2024-04-18

**Authors:** Chao Liu, Changjin Li, Teng Zhao, Manli Yu, Xinmiao Huang, Jiang Cao, Songqun Huang, Zhifu Guo

**Affiliations:** Department of Cardiology, Changhai Hospital, 168 Changhai Road, Shanghai 200433, China; Department of Cardiology, Changhai Hospital, 168 Changhai Road, Shanghai 200433, China; Department of Cardiology, Changhai Hospital, 168 Changhai Road, Shanghai 200433, China; Department of Cardiology, Changhai Hospital, 168 Changhai Road, Shanghai 200433, China; Department of Cardiology, Changhai Hospital, 168 Changhai Road, Shanghai 200433, China; Department of Cardiology, Changhai Hospital, 168 Changhai Road, Shanghai 200433, China; Department of Cardiology, Changhai Hospital, 168 Changhai Road, Shanghai 200433, China; Department of Cardiology, Changhai Hospital, 168 Changhai Road, Shanghai 200433, China

**Keywords:** Cryoballoon, Left atrial appendage electrical isolation, LAA flow velocity, Thrombus events

## Abstract

**Aims:**

Left atrial appendage electrical isolation (LAAEI) has demonstrated a significant enhancement in the success rate of atrial fibrillation (AF) ablation. Nevertheless, concerns persist about the safety of LAAEI, particularly regarding alterations in left atrial appendage (LAA) flow velocity and the potential risks of thrombus. This study aimed to assess the efficacy and safety of LAAEI, investigating changes in LAA flow velocity in canines.

**Methods and results:**

The study comprised a total of 10 canines. The LAAEI procedure used by a 23 mm cryoballoon of the second generation was conducted at least 180 s. Intracardiac ultrasonography (ICE) was employed to quantify the velocity flow of the LAA both prior to and following LAAEI. Following a 3-month period, subsequent evaluations were performed to assess the LAA velocity flow and the potential reconnection. Histopathological examination was conducted. Left atrial appendage electrical isolation was effectively accomplished in all canines, resulting in a 100% acute success rate (10/10). The flow velocity in the LAA showed a notable reduction during LAAEI as compared with the values before the ablation procedure (53.12 ± 5.89 vs. 42.01 ± 9.22 cm/s, *P* = 0.007). After the follow-up, reconnection was observed in four canines, leading to a success rate of LAAEI of 60% (6/10). The flow velocity in the LAA was consistently lower (53.12 ± 5.89 vs. 44.33 ± 10.49 cm/s, *P* = 0.006), and no blood clot development was observed. The histopathological study indicated that there was consistent and complete injury to the LAA, affecting all layers of its wall. The injured tissue was subsequently replaced by fibrous tissue.

**Conclusion:**

The feasibility of using cryoballoon ablation for LAAEI was confirmed in canines, leading to a significant reduction of LAA flow velocity after ablation. Some restoration of LAA flow velocity after ablation may be linked to the passive movement of the LAA and potential reconnecting. However, this conclusion is limited to animal study; more clinical data are needed to further illustrate the safety and accessibility of LAAEI in humans.

## Graphical Abstract

**Graphical Abstract:**
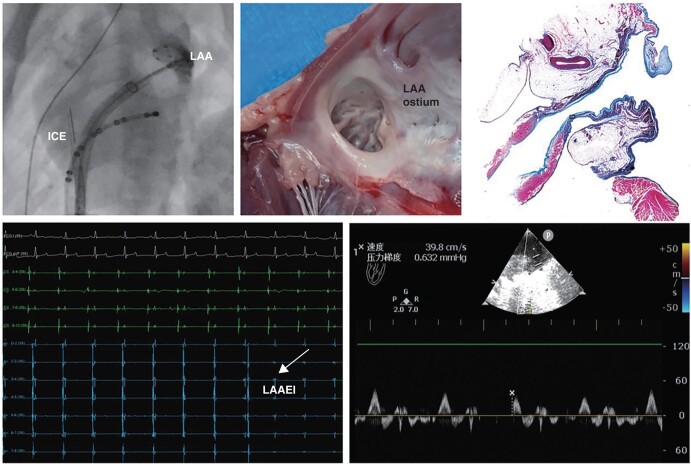
Our study represents an infrequent and novel animal experiment to confirm the safety and efficacy of the cryoballoon (CB) ablation for left atrial appendage electrical isolation (LAAEI) in canines. Although second-generation cryoballoon (CB2)-induced LAAEI is a feasible technique in canines, the LAA velocity flow often decreases after ablation, and some recovery of LAA flow velocity is mostly linked to the passive movement of the LAA and left atrial, as well as the possibility of potential reconnection. The intracardiac ultrasonography (ICE)-guided transseptal puncture obtained the effective and safe outcomes in our canine study and substantially reduced X-ray exposure.

**What’s new?**

- Our study represents an infrequent and novel animal experiment to explore the safety and efficacy of the cryoballoon (CB) ablation for left atrial appendage electrical isolation (LAAEI) in canines.
- Although second-generation cryoballoon (CB2)-induced LAAEI is a feasible technique in canines, the LAA velocity flow often decreases after ablation, and some recovery of LAA flow velocity is mostly linked to the passive movement of the LAA and left atrial, as well as the possibility of potential reconnection.
- The intracardiac ultrasonography (ICE)-guided transseptal puncture obtained the effective and safe outcomes in our canine study and substantially reduced X-ray exposure.

## Introduction

Since Haïssaguerre *et al.*^1^ confirmed that the pulmonary vein (PV) is the main trigger area of atrial fibrillation (AF), PV isolation (PVI) has been considered the fundamental procedure for catheter ablation of AF. Although PVI is an established therapy for paroxysmal AF, it is not effective for long-standing persistent AF.^2^

Multiple studies emphasize the importance of ectopic triggers located beyond the PVs in relation to atrial fibrillation. These triggers can be found in structures such as the superior vena cava (SVC), ligaments of Marshall (LOM), coronary sinus (CS), and the posterior left atrial (LA) wall.^2–5^ Notably, the LA appendage (LAA) is identified as an inconspicuous yet crucial ectopic focus.

A prior large-scale study revealed that the LAA’s involvement in triggering AF can be as high as 27%,^2^ underscoring its importance as a proarrhythmic substrate for AF. The recurrence rate of AF in the LAAEI ablation group, as evidenced by a 1-year follow-up, was significantly lower than in other control groups, affirming the beneficial impact of LAAEI on AF prognosis. A meta-analysis further suggested a substantial reduction of 22.9% in absolute recurrence rates when employing a combined PVI and LAAEI strategy compared with PVI alone,^6^ particularly for persistent AF. However, concerns about the safety of LAAEI persist, with a focus on its impact on LAA function, as well as the potential risk of thrombotic events and stroke. The BELIEF study confirmed a reduction in LAA emptying flow velocity following LAAEI without an increased incidence of stroke events.^7^ In contrast, Kim *et al.*^8^ observed a significant increase in stroke risk after LAAEI, despite no decrease in LAA flow velocity. Although the latest study by Yorgun *et al.*^9^ also confirmed the efficacy of LAAEI, it also highlighted concerns about thromboembolic complications (7.9%) after LAAEI. Consequently, the safety of LAAEI remains a subject of ongoing debate.

At present, radiofrequency (RF) ablation and cryoballoon (CB) ablation are two prevalent techniques used in catheter ablation.^10^ Radiofrequency ablation is a procedure that creates straight lesions by applying RF energy point by point. On the other hand, CB ablation creates circular lesions by using N_2_O to freeze the tissue around it. The traditional LAAEI approach entails performing RF ablation in a meticulous manner, targeting each individual site around the LAA ostium. Nevertheless, the noteworthy drawback lies in the potential risk of the catheter penetrating into the LAA, resulting in cardiac tamponade. This risk arises from the uneven thickness and crypts present on the LAA ostium. Furthermore, the damage caused by ablation in a point-by-point manner is typically uneven, which raises the chances of potential reconnection in the LAA. Given these constraints, RF ablation for LAAEI is considered unfeasible.

In recent years, CB ablation has gained prominence as a novel technique in AF ablation.^11,12^ Utilizing the CB to block PVs and releasing N_2_O to induce annular damage, CB ablation boasts advantages such as time efficiency, stable attachment, and uniform damage. Leveraging these advantages, CB ablation is theoretically considered more efficient than RF ablation for LAAEI, potentially emerging as an ideal LAAEI technology in the future. Although some clinical studies^13–15^ have confirmed the feasibility and safety of CB ablation for LAAEI, the overall sample size remains limited, warranting further research.

In our study, we selected healthy canines with sinus rhythm to assess the efficacy and safety of the second-generation CB for LAAEI. Our focus extends to evaluating the impacts on LAA flow velocity and systolic function, aiming to provide robust evidence supporting the feasibility of combining PVI with LAAEI for persistent AF ablation in clinical treatment.

## Methods

**Figure 1:**
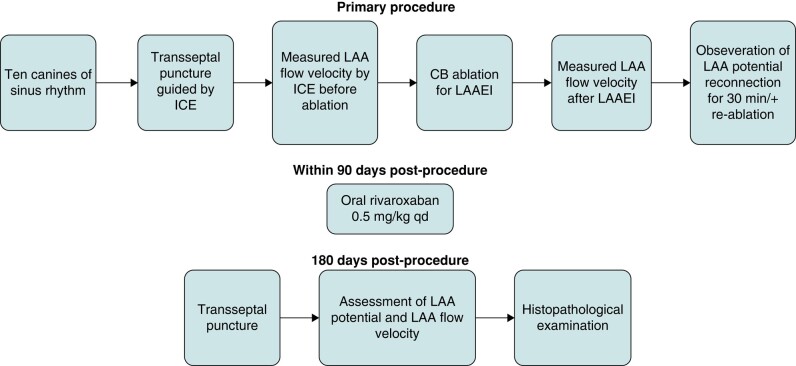
The research protocol. ICE, intracardiac echocardiography; LAA, left atrial appendage; LAAEI, left atrial appendage electrical isolation; CB, cryoballoon.

Before the formal study, three canines were selected for preliminary experiment; the whole experimental process was simulated, and CB ablation for LAAEI under open-chest direct vision to observe the range of frost and LAA activity was performed. *Figure 1* shows the steps of the research protocol, the following sections being the details.

### Animal

Thirteen healthy, male beagle canines (13–15 kg) were included. None received anticoagulant therapy and were routinely fasted for at least 8 h before ablation.

All animal research was performed in accordance with the Animal Research: Reporting of In Vivo Experiments (ARRIVE) guidelines and approved by China’s ethics committees of Changhai Hospital before the initiation of the study (ethics number: CHEC(A.E) 2022-014).

### Vascular puncture

The ablation procedures were performed by the guidance of NavX three-dimensional mapping system under general anaesthesia. Invasive arterial blood pressure, oxygen saturation, and electrocardiogram (ECG) were continuously monitored in perioperative period. The left femoral vein and right femoral vein/artery were respectively punctured with Seldinger’s method. A 6 Fr steerable decapolar catheter was delivered to the coronary sinus. A 10 Fr sheath was placed in the left femoral vein, the guidewire (0.032 in, 180 cm Super Stiff, St. Jude Medical, St. Paul, MN, USA) was delivered to the SVC via the right femoral vein, and a 7 Fr sheath was placed in the right femoral artery.

### Transseptal puncture

**Figure 2:**
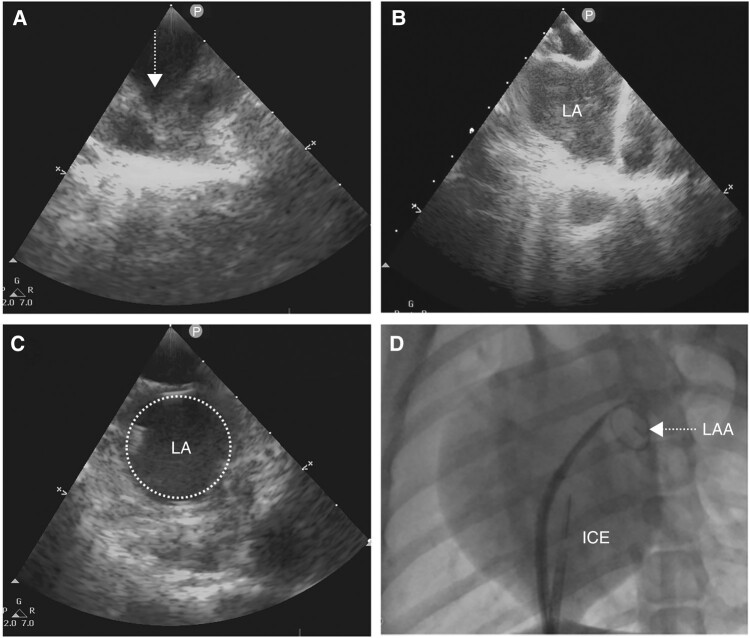
The atrial septal puncture under the guidance of ICE. (*A*) The ICE image showed RA, LA, and atrial septum, and the puncture sheath delivered forward against the atrial septum to form the ‘tent sign’. (*B*) Puncture the needle into the LA smoothly; saline injection can be seen ‘bubble sign’. (*C*) The steel wire was sent along the sheath tube and coiled; the LA was full of ‘ring’. (*D*) The circular guidewire was located in the LAA under fluoroscopy. ICE, intracardiac echocardiography; RA, right atrial; LA, left atrial; LAA, left atrial appendage.

Transseptal puncture by modified Brockenbrough method (BRK-1, St. Jude Medical) was performed under the guidance of intracardiac ultrasound (ICE). Intracardiac ultrasound catheter was delivered through the 10 Fr sheath to the high right atrium (RA) with 1 o’clock direction under fluoroscopy. Adjust the P-bend of the catheter and rotate slowly clockwise to scan. The RA, LA, and atrial septum appeared successively later. Fix the ICE, rotate clockwise, and withdraw the SL1 transseptal sheath (8.5 F SL1 transseptal sheath, St. Jude Medical) towards the atrial septum; the ‘tent sign’ occurred (*Figure 2A*). When the BRK needle completed transseptal puncture, the ‘tent sign’ disappeared. Inject the heparin normal saline, and the ‘bubble sign’ appeared (*Figure 2B*), indicating that LA access was established (*Figure 2C*). Deliver the SL1 sheath forward to the LA, exchange guidewire, and withdraw the SL1 sheath. Confirm safety of cardiac shadow under fluoroscopy (*Figure 2D*), and heparinization was administered intravenously at 0.6 mg/kg.

### Left atrial appendage flow velocity measurement

The ICE catheter was manipulated to assess the presence of thrombus in LAA, and the pre-ablation LAA flow velocity was recorded when the waveform was stable. Left atrial appendage flow velocity reserve was defined as >0.4 m/s, and LAA flow velocity decrease was defined as <0.4 m/s.

### Cryoballoon ablation for left atrial appendage electrical isolation

**Figure 3:**
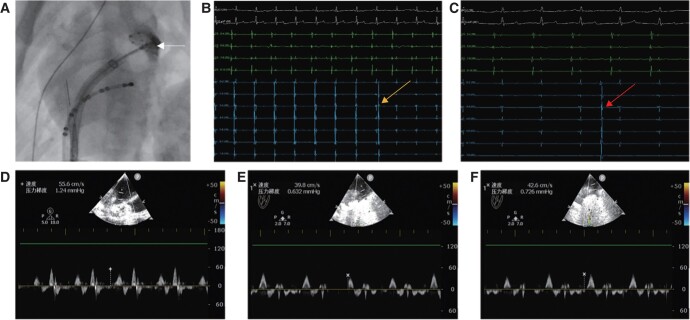
The procedure of LAAEI and the measure of LAA velocity flow during ablation. (*A*) The cryoballoon successfully blocked the LAA, and the contrast agent was injected to show no reflux. (*B*) Left atrial appendage potential dropped during CB ablation (arrow showed). (*C*) At 30 min after ablation, LAA autorhythmicity potential was detected (arrow showed), reconfirming successful LAAEI. (*D*–*F*) Left atrial appendage velocity measured before and after ablation and follow-up. CB, cryoballoon; LAAEI, left atrial appendage electrical isolation.

Once LA access was established, the 12 Fr steerable transseptal sheath (FlexCathVR, Medtronic CryoCath, Minneapolis, MN, USA) was placed into the LA by the guidewire, and LAA angiography was performed under RAO 20° and CAU 20° of fluroscopy. The Achieve ^TM^ (Medtronic, Minneapolis, MN, USA) circular mapping catheter and the second-generation 23 mm CB catheter (Arctic Front Advance^TM^ and Arctic Front Advance^TM^ ST, Medtronic, Minneapolis, MN, USA) were delivered to the LA through the steerable sheath. When the circular mapping catheter was inserted to the LAA, the CB was inflated later and manoeuvred towards to occlude the LAA ostia. Perfect occlusion criterion was defined as complete filling of the LAA and no backflow of the LA, while the contrast agent was injected through the steerable transseptal sheath, the CB ablation began later (*Figure 3A*). Basic freezing dose was 180 s and can be adjusted according to LAA isolation time during the ablation, as follows: time to isolation (TTI) < 90 s ablation dose 180 s, TTI = 90–120 s plus 60 s ablation dose, and TTI = 120–150 s plus 120 s ablation dose; the maximum dose was not more than 300 s (*Figure* *3B* and *C*). The monitoring of the left phrenic nerve (LPN) injury and pericardial tamponade was performed by intermittent abdominal and cardiac fluoroscopy throughout the CB ablation. In addition, close observation of intracardiac ECG is also essential. While the conditions as sinus bradycardia, junctional heart rhythm, ST-segment changes of ECG, freezing temperature falling below −60°, and cardiac or diaphragmatic activity drops occurred during the process, the CB ablation was stopped immediately, and the CB was readjusted for re-ablation. Coronary angiography was performed immediately after completion of LAAEI to exclude left circumflex (LCX) spasm. The endpoint of CB ablation was acute LAEEI, defined as the complete disappearance of the LAA potential observed from the circular mapping catheter, or spontaneous potentials unrelated to the LA occurred. The data of LAA flow velocity, time to LAAEI, temperature of LAAEI, and total freezing dose were recorded in procedures.

For the detection of early potential reconnection, an observation period of 30 min should be allowed, and CB ablation was performed once more in canines with potential reconnection. The LAA flow velocity was measured by ICE catheter later, all sheath tubes were removed, and the procedure was ended (*Figure 3D–F*).

### Post-procedural management and follow-up

Rivaroxaban was given orally 0.5 mg/kg daily within 3 months. About 180–200 days of follow-up, routine transseptal puncture was performed to assess LAA potential block using the Achieve circular mapping catheter. The ICE catheter was used to measure LAA thrombus and record LAA flow velocity.

### Histological analysis

All canines were sacrificed by intravenous injection of 10% potassium chloride, and the primary organs including the heart, lung, kidney, and spleen were dissected and analysed by pathology to obtain evidence of infarction. The LA was dissected to observe the extent of LAA damage, and the relationship with LAA potential reconnection was explored. The LAA and surrounding 5 mm tissue were excised and fixed with formalin solution. After embedding in paraffin, HE or Masson staining was performed to observe the degree of fibrosis and surrounding tissue damage in the LAA ablation region.

### Statistical analysis

The experimental data were analysed by SPSS (version 25.0) software. The measurement data were expressed in the form of *x* ± *s*, with *t*-test or rank sum test. Counting data were expressed as percentage (%), with χ^2^ or Fisher test. *P* value < 0.05 was considered statistically significant.

## Results

Left atrial appendage electrical isolation was effectively performed in all canines. The transseptal puncture took an average of 10.70 ± 3.89 min, the freezing dose was 210.00 ± 42.43 s, the time to LAAEI was 60.80 ± 47.04 s, and the minimum freezing temperature reached was −57.60 ± 6.04°C. *Table 1* displays the procedural specifics.

**Table 1:** Detailed data of 10 experimental canines

Number	Transseptal time (min)	Total freezing bonus (s)	Time to isolation (s)	Lowest temperature (°C)	LAA flow velocity (cm/s)			LAA electric potential			Follow-up period (day)
					Pre-cryo	Immediately after cryo	Follow-up	Pre-cryo	Immediately after cryo	Follow-up	
1	8.5	180	40	−56	58.5	59.8	58.7	Exist	Isolation	Isolation	191
2	7	180	14	−61	55.6	39.8	42.6	Exist	Isolation	Isolation	191
3	12.5	180	70	−62	45.4	48.6	35.8	Exist	Isolation	Reconnection	180
4	15.5	180 + 60	102	−44	61	40	58.7	Exist	Isolation	Reconnection	180
5	6	180	10	−66	49	39.3	34	Exist	Isolation	Isolation	180
6	13	180	18	−56	47.3	51.1	43.8	Exist	Isolation	Reconnection	180
7	15	180 + 120	142	−56	58.7	44.5	42.8	Exist	Isolation	Isolation	198
8	15	180 + 60	102	−63	56.1	34.8	36.6	Exist	Isolation	Isolation	198
9	8.5	180 + 60	92	−56	45.2	28.8	32.3	Exist	Isolation	Isolation	198
10	6	180	18	−56	54.4	33.4	58	Exist	Isolation	Reconnection	191

LAA, left atrial appendage.

### Left atrial appendage electrical isolation

Left atrial appendage electrical isolation was successfully performed in all 10 canines, with a 100% acute success rate. This included six canines treated with freezing doses of 180 s, three canines with doses of 240 s, and one canine with a dose of 300 s. After a follow-up period of 188.70 ± 8.02 days, six canines maintained their status of LAAEI, while four canines experienced potential reconnection of the LAA. The 6-month success rate of CB ablation for LAAEI was 60%.

### Left atrial appendage flow velocity and thrombus

Following LAAEI, a significant reduction in LAA flow velocity was observed compared with pre-ablation values (53.12 ± 5.89 vs. 42.01 ± 9.22 cm/s, *P* = 0.007). This lower velocity persisted at follow-up (53.12 ± 5.89 vs. 44.33 ± 10.49 cm/s, *P* = 0.006). No thrombus formation was detected within the LAA before ablation, and this remained unchanged after the 188.70 ± 8.02 days follow-up.

### Transseptal puncture

Transseptal puncture, guided by the ICE catheter, was successfully performed in canines with a 100% success rate. Out of the total of 10 canines, 9 were pierced successfully on the initial try, while 1 required two changes in the angle of puncture. The mean duration for transseptal puncture was 10.70 ± 3.89 min.

### Perioperative complications

**Figure 4:**
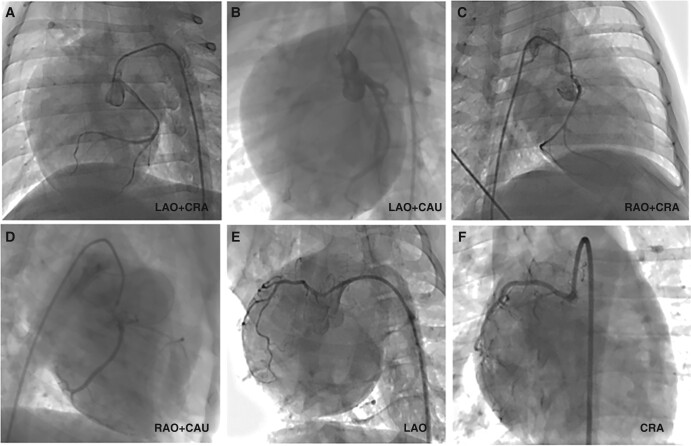
Coronary angiography on canines after cryoballoon ablation. All the canines underwent coronary angiography immediately after CB ablation at six angiography angles (*A*–*F*), and no abnormal findings or LCX spasm events were observed. LCX, left circumflex. LAO, left anterior oblique; RAO, right anterior oblique; CRA, cranial; CAU, caudal.

In the preliminary experiment, we attempted to perform CB ablation for LAAEI using direct visualization after thoracotomy. Our goal was to examine the extent of frost formation during CB ablation, in order to acquire more visually clear and precise data. The LPN is observable ambulating on the surface of the LAA under direct visual examination. The radiography demonstrates the CB being blocked at the LAA opening and initiating CB ablation, resulting in frost formation over the atrioventricular groove. During CB ablation, the researchers noticed a dynamic evolution of LAA activities, which transitioned from active to passive systolic and diastolic motions. During the perioperative period, a canine patient exhibited sinus bradycardia while being induced under anaesthesia. The condition was rapidly treated by administering 1 mg of atropine intravenously. Another canine showed reduced diaphragmatic activity while undergoing CB ablation, requiring the freezing process to be stopped immediately. The canine recovered within around 3 min. A single canine experienced a post-operative inguinal haematoma, which did not worsen after stopping oral anticoagulant medication for 5 days. Therefore, the decision was made to continue with anticoagulation treatment. There was no evidence of pericardial tamponade, and the coronary angiography did not show any spasm in the LCX artery (*Figure 4A–F*).

### Gross anatomy

**Figure 5:**
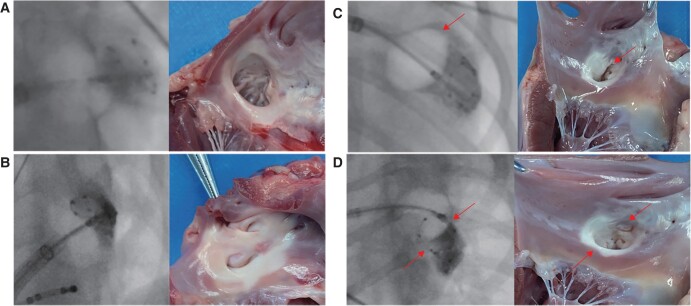
Comparison of left atrial appendage anatomy and imaging. (*A*) No. 2 canine: the freezing dose was 180 s, TTI was 14 s, and the LAAEI state was maintained during follow-up. (*B*) No. 7 canine: the freezing dose was 300 s, TTI was 142 s, and the LAAEI state was maintained during follow-up. (*C*) No. 3 canine: the freezing dose was 180 s, TTI was 70 s, the LAA potential reconnection occurred during follow-up, and a defect in the injury region is discernible at the 12 o’clock position of the LAA ostium. (*D*) No. 10 canines were treated with freezing dose of 180 s and isolation time of 18 s. Left atrial appendage potential reconnection was reviewed after follow-up, and the injury region defects were at both the 12 and 6 o’clock positions of the LAA ostium. TTI, time to isolation.

Upon dissecting the LAA to examine the extent of injury regions, depicted in *Figure* *5A* and *B*, a stable and uniformly extensive scar is evident on the LAA ostium, as illustrated in *Figure 5C*. Notably, a defect in the injury region is discernible at the 12 o’clock position of the LAA ostium (orientation determined by the line from the mitral annulus chordae bundle to the LAA ostium, with the 12 o’clock above and the 6 o’clock below).

*Figure 5D* further illustrates the injury region defects at both the 12 and 6 o’clock positions of the LAA ostium. The injury regions in *Figure* *5C* and *D* appear smaller than those observed in *Figure* *5A* and *B*. This dissection provides a detailed visual representation of the scar formation and injury region defects.

### Histopathological analysis

**Figure 6:**
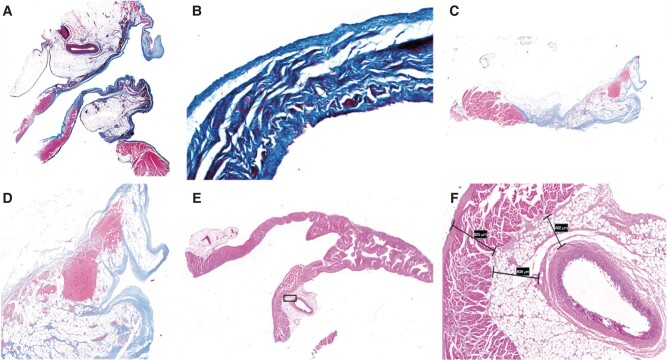
Pathological anatomy of the left atrial appendage. (*A*) LAAEI canines: LAA was incised longitudinally and showed clear delineation of the damage zone. (*B*) Freezing damage reached through the wall of the LAA ostium (Masson stain 10×). (*C*) Left atrial appendage potential reconnection canines: clear boundary was observed between the normal tissue of the LAA and the damage zone. (*D*) Freezing damage not permeable wall of the LAA ostium (Masson stain 10×). (*E*) The anatomy showed that the LAA ostium was adjacent to the LCX. (*F*) Freezing damage did not involve LCX (HE staining 10×). LAAEI, left atrial appendage electrical isolation; LAA, left atrial appendage; LCX, left circumflex.

The histopathological examination of the LAA in canines with LAAEI revealed a continuous injury zone. The depth of the lesion was consistent and extended through the entire wall of the LAA ostium. The affected area was entirely replaced by fibrous connective tissue, as shown in *Figure* *6A* and *B*. In canines with the potential for reconnection in the LAA, microscopic examination revealed that the injury region was not continuous or uniform. There was still normal myocardial tissue present alongside the injury region, indicating that it had not been completely replaced by fibrous connective tissue. Furthermore, the depth of the lesion did not extend through the entire thickness of the LAA ostium (*Figure* *6C* and *D*). Furthermore, there was no evidence of LCX injury when examined under a microscope (*Figure* *6E* and *F*).

## Discussion

This study represents the inaugural animal experiment investigating second-generation cryoballoon (CB2) ablation for LAAEI in canines. The main results of the investigation indicate that (i) the current CB2 ablation, which involves a duration of 180 s plus additional adjustment, is a feasible, effective, and safe technique for LAAEI. (ii) A basic freezing duration of 180 s causes damage across the LAA ostium, making it easier to achieve long-lasting LAAEI with optimal LAA closure. (iii) The velocity of blood flow in the LAA often decreases after being affected by CB2-induced LAAEI. The restoration or recovery of LAA flow velocity is mostly linked to the passive movement of the LAA during the contraction and relaxation phases, as well as the possibility of reconnection.

### The efficacy of left atrial appendage electrical isolation

The LAA, originating from the primitive LA during the embryonic period, is formed by the muscular sleeves of the PV extending outward from the posterior wall of the LA.^16^ Histologically similar to PVs, the LAA is recognized as the ‘fifth PV’ and is a crucial arrhythmogenic substrate in AF.^17^ A meta-analysis has reported that several studies confirmed LAAEI effectively reduces the 22.9% recurrence rate of AF.^6^ The use of RF energy for gradual point ablation around the LAA ostium seems to be challenging, and complications such as cardiac tamponade are not uncommon.^10^

Currently, CB ablation has gained wide acceptance in AF ablation due to its effective procedure, uniform injury, and lower incidence of complications such as vascular stenosis, inguinal haematoma, pseudoaneurysm, and pericardial tamponade. Cryoballoon ablation is anticipated to be an ideal technique for LAAEI.^9,13,18^ Studies by Yorgun *et al.*,^13^ Bordignon *et al.*,^14^ and Chen *et al.*^15^ have demonstrated the feasibility of CB ablation for LAAEI, although comprehensive studies on the efficacy and feasibility are still lacking, necessitating further clarification.

Effective ablation time is a major concern in CB ablation. Su *et al.*^19^ and Wei *et al.*^20^ have shown that freezing doses of 90 s can be effective for PV and SVC isolation, respectively. Nevertheless, due to its muscular nature, the LAA necessitates a higher dosage of freezing in order to cause damage throughout its entire thickness. Utilizing prior research, we devised a procedure that includes a standard freezing duration of 180 s, along with supplementary freezing doses based on the duration needed for LAA isolation during ablation. The findings of our study indicate that the utilization of the 23 mm CB2 to block the LAA ostium is highly successful in isolating the LAA potential. Each of the 10 canines exhibited LAA potential and successfully attained LAAEI, resulting in a 100% acute success rate. This rate is notably superior to the 89% acute success rate under RF energy shown in animal trials conducted by Panikker *et al.*^21^ Over the course of the average follow-up period of 188.7 ± 8.02 days, six canines consistently maintained their LAAEI status. However, four canines experienced a potential reconnection of the LAA, resulting in a success rate of LAAEI at 6 months of 60%. The efficiency of LAAEI is determined by two essential factors: the freezing dose and the occlusion scenario. Our investigation found no significant disparity in the 6-month success rate of LAAEI among canines with varying freezing dosages. The histopathological findings further verified that CB ablation effectively induced full-thickness damage surrounding the LAA with a freezing dose of 180 s. Hence, a duration of 180 s at freezing temperatures seems to be adequate for achieving LAAEI when optimal closure of the LAA is achieved.

In order to better examine the potential reconnection of the LAA, we undertook a thorough analysis of the anatomical structure and data from canines. The evaluation revealed that canines with LAAEI displayed a consistent and uniform lesion at the opening of the LAA. Left atrial appendage angiography showed that the CB2 was delivered superiorly towards the LAA, covering the LAA ostium completely and uniformly. The ablation function domain of the CB2 was in the anterior hemisphere, aligning with the structure of the CB2 and promoting homogeneous and stable lesions.

Histopathological results confirmed the depth of LAA lesions to be transmural, replaced by fibrous tissue. In canines with LAA potential reconnection, varying extents of scar defects were observed at the 12 and 6 o’clock positions. The CB2, visible on imaging, exhibited a ‘hockey stick’ sign, indicating reverse occlusion. This technique increased tension on one side of the CB2, causing unstable occlusion on the other side and, consequently, avoiding the ablation domain.

In canines with scar defects at the 12 o’clock position of the LAA ostium (*Figure 5C*), the occluded area may extend over the anterior hemisphere of the CB2, potentially causing insufficient ablation. For canines with scar defects at both the 12 and 6 o’clock positions of the LAA ostium (*Figure 5D*), imaging revealed that the hemisphere below the CB2 predominantly occluded the LAA ostium. The anterior end of the CB2 entered the LAA ostium and closely adhered to the superior border, while the inferior part of the LAA reached the edge of the ablation domain. This situation may also pose challenges in ensuring adequate ablation of the LAA. Histopathological examination demonstrated discontinuous and non-transmural injuries, with some normal myocardial tissue not completely replaced by fibrous tissue. Therefore, we observed that segmental ablation could be considered in cases of difficult occlusion, ensuring as circular an isolation of the LAA as possible.

Panikker *et al.*^21^ reported that the superior and inferior parts of the LAA ostium were thicker and posed challenges for achieving sufficient RF ablation, resulting in LAA reconnection. Chen *et al.*^15^ also attempted to improve contact with the caudal segment of the LAA ostium through the ‘pull-down’ method with CB2 in humans. Therefore, the anatomical factor of LAA should also be taken into consideration. While we observed a similar phenomenon, when combined with pathological results, it was found that both the superior and inferior aspects of LAA lesions reached the transmural level, indicating that the freezing dose was sufficient. Hence, we concluded that the LAA occlusion situation with CB2 was closely related to the long-term success rate of LAAEI. It is noteworthy that the location of transseptal puncture significantly influences LAA occlusion with CB2. In this study, canine heart anatomy differs somewhat from humans, making it challenging to precisely determine the optimal location for atrial septal puncture. Nevertheless, electrophysiologists, with their extensive experience in human transseptal puncture, may achieve a higher success rate with CB ablation of LAAEI.

### The safety of left atrial appendage electrical isolation

The CB ablation treatment for LAAEI is reasonably convenient; nonetheless, certain problems, including pericardial tamponade, LPN injury, LCX spasm, thrombus formation, and stroke events, have been documented.^9,13,18^ No occurrences of pericardial tamponade or LAA rupture were detected in our study. Ensuring careful manipulation and firm positioning of the circular mapping catheter, while maintaining low tension, was crucial in preventing rupture of the LAA during the delivery of the CB2 for occlusion of the LAA ostium. The documented occurrence of LCX spasm following LAAEI with CB ablation is estimated to be around 4%.^13^ Canpolat *et al.*^22^ also documented an instance of LCX spasm after LAAEI. This muscle contraction is frequently linked to the formation of ice on the neighbouring LCX artery during CB ablation. Nevertheless, our study did not find any ST-segment changes on the ECG or LCX spasm throughout the surgery.

In terms of anatomy, the LPN passes over the LAA surface,^23^ and LAA ablation increases the potential risk of LPN injury. Romero *et al.* performed LPN mapping on 66 patients during the LAA ablation procedure and verified that around 74% of LPN travelled across the middle and far sections of the LAA, while only 4.5% were found at the LAA ostium. During this trial, we observed only one canine experiencing a slight decrease in diaphragmatic activity. This indicates that doing ablation in close proximity to the LAA opening could successfully prevent harm to the LPN. During this study, we observed that only one canine had a minor decrease in diaphragmatic activity. This decrease was temporary and lasted for ∼2 min after stopping the ablation. We believe that this decrease was caused by the low temperature of the ablation in the carotid body. Romero *et al.* recommended the strategy of segmented and linear LA ablation for patients in whom the LPN successfully passes at the LAA ostium. Nevertheless, this approach may compromise the systolic function of the entire left atrium, thereby heightening the potential susceptibility to thrombosis and stroke.^8,24^ Despite previous studies^13,18^ documenting the complications of LPN injury in CB ablation of LAAEI, our study, combined with these findings, supports the notion that CB ablation is a reasonably safe approach for achieving LAAEI by targeting the LAA ostium. This approach effectively minimizes the risk of LPN injury. Nevertheless, there is a lack of studies directly comparing the safety and efficacy of CB ablation and RF ablation for LAAEI. Further clinical research is necessary to validate these findings.

Recent multicentre retrospective analysis has referred that LAA ablation could disrupt the connection between electrical and mechanical functions, resulting in a decrease in LAA flow velocity and an elevated risk of thrombus formation or stroke.^9^ In these comprehensive study on catheter ablation of LAAEI, Yorgun *et al.*^9,13,18^ found that despite a noticeable decrease in LAA flow velocity during follow-up, some of patients still had a flow velocity > 0.4 m/s. This could be attributed to various factors, including potential reconnection, spontaneous electrical activity in the LAA, or the influence of left ventricular diastole. Importantly, no instances of LAA thrombosis were observed in patients with reduced LAA flow velocity at the 12-month follow-up. Nevertheless, Kim *et al.*^8^ found that the increased stroke risk was independent of LAA flow velocity after LAAEI. Consequently, there is a debate regarding whether there is a higher likelihood of stroke following LAAEI, and additional evidence is needed to clarify the alterations in LAA flow velocity and the associated mechanisms.

Our findings demonstrated a notable decrease in LAA flow velocity following LAAEI and subsequent evaluation. Additionally, in four canines, the LAA flow velocity (>0.4 m/s) remained unchanged. Below, we have examined the relevant factors that have an impact. Prior to evaluating the LAA flow velocity, the potential isolation of the LAA was reaffirmed in all canines using a circular catheter. Therefore, the possibility of reconnection was not taken into account. Upon reviewing all intracardiac electrograms during the procedures, we did not detect any consistent spontaneous electrical activity in the LAA. However, we did observe a consistent and stable waveform in the ultrasound image of the LAA flow velocity. Therefore, we can conclude that the preserved flow velocity is not due to any spontaneous activity in the LAA.

For this study, we specifically chose canines that were in good health and had a normal heart rhythm. We excluded canines with left atrium remodelling, which would have made them different from the AF canine model. Ablation of the LAAEI was carried out using direct visualization during thoracotomy in a pre-experimental setting. The real-time observation revealed a gradual decline in the strength of the LAA contractions, ultimately resulting in passive motion along with the LA. Consequently, the maintenance of LAA flow velocity following ablation was primarily attributed to the passive movement associated with the LA.

After a follow-up period of 188.70 ± 8.02 days, the LAA flow velocity improved in two out of the six canines that initially had reduced LAA flow velocity. Later, the reconnection of LAA potential was confirmed using a circular mapping catheter. The remaining four canines continued to exhibit reduced levels of LAA flow velocity. The status of LAA potential isolation was detected in all cases, confirming the association between LAA potential reconnection and the recovery of flow velocity. Hence, the primary factors taken into account for the conservation of LAA flow velocity were as follows^13,15,18^: (i) inferior levels of LA remodelling and passive motion of LAA accompanied with systolic and diastolic of LA and (ii) LAA potential reconnection, which was consistent with Yorgun *et al.*’s research.

It is worth noting that we had observed a significantly weakened of LAA contraction until passive motion accompanied with LA during CB ablation in pre-experiment. For AF patients with inferior LA remodelling, preservation of LAA flow velocity is completely possible even though LAA systolic function absolutely dropped. Based on this phenomenon, it seems a stretch to interpret preserved LAA velocity flow as retained LAA function. Although Di Biase *et al.*^25^ and Fink *et al.*^26^ pointed to the reduced LAA flow velocity as an independent predictor of stroke, Kim *et al.*^8^ similarly confirmed that the increased risk of stroke was not associated with LAA velocity flow after LAAEI. As a result, whether LAA flow rate can be a basis for post-operative guidance of oral anticoagulation (OAC) or LAA closure (LAAC) may still require further research to demonstrate.

Previous studies reported no increased risk of stroke events after LAAEI, whereas Kim *et al.*^8^ and Rillig *et al.*^24,27^ concluded the opposite view in their study, and it is worth mentioning that linear ablation of LA to passive LAAEI was employed. Hence, the utilization of various approaches for LAAEI may also entail an increased likelihood of thrombosis and stroke occurrences. All canines in our study were administered rivaroxaban for a duration of 3 months following CB ablation of LAAEI. During the follow-up period, no instances of LAA thrombosis or stroke events were observed. Additionally, histological examination revealed the absence of any noticeable infarct foci on vital organs. Our analysis of the causes behind these results is as follows: (i) the experiment canines had no history of AF and abnormal LA contraction and lacked conditions for thrombosis; (ii) compared with the strategy of extensive linear ablation of LA, CB ablation was geared to the active LAAEI strategy and did not impair the LA contraction; and (iii) the number of samples was small, and no control group of non-OAC was installed. Hence, it is possible that our results may contain some prejudice. However, in our investigation, we did not observe any instances of blood clot formation or stroke in canines following the ablation of the LAAEI combined with OAC. This confirms that the procedure is both feasible and safe. When comparing the passive strategy of LAAEI with the active ablation strategy, it is possible that the active strategy may have a lower risk of thrombosis. However, there are currently no clinical studies that directly compare RF ablation with CB ablation or the active strategy with the passive strategy of LAAEI. Therefore, further investigation is needed to determine which type of ablation strategy is preferable.

### Transseptal puncture under the guidance of intracardiac ultrasonography

In animal experiments of cardiovascular intervention, atrial septal puncture is an important technique to establish the access between LA and RA. Due to the disparity in heart anatomy between canines and humans, it is challenging to perform atrial septal puncture under fluoroscopy during preliminary experiments. Intracardiac echocardiography provides a clear visualization of the anatomy within the cardiac chamber and offers precise guidance during the procedure.^28^ Consequently, the atrial septal puncture was performed with the assistance of ICE in the subsequent investigation.

In our study, the ICE-guided transseptal puncture guided was successfully performed in canines with a 100% success rate and no complications occurred. This technique yielded effective and reliable outcomes in our study, effectively preventing occurrences of cardiac tamponade and substantially reducing the duration of radiation exposure.

### Study limitations

There are various constraints in our study. At first, the number of canines included in the study was restricted. Nevertheless, it showcased the practicability of CB ablation for LAAEI and the alterations in LAA velocity flow. Another limitation in this study is the lack of a control group, which means that factors such as freezing dose, OAC, or non-OAC use were not taken into account. This raises questions about whether the negative thrombus events were caused by the safety of the LAAEI strategy of CB ablation itself or the potential risk reduction due to OAC. Furthermore, the absence of canines with AF in our study is another limitation. Currently, it is not feasible to create a persistent AF model using canines. Choosing canines that are in good health and have a normal heart rhythm, while excluding those with atrial fibrillation and LA remodelling, is beneficial for studying the relationship between changes in LAA flow velocity and LAAEI through catheter ablation. However, it is crucial to recognize that this study is conducted on animals, and the validity of its findings ultimately depends on the confirmation from extensive clinical studies with a large number of participants.

## Conclusions

Under a freezing dose of 180 s, CB ablation can effectively achieve circular transmural injury to the LAA ostium, with a 100% (10/10) success rate for acute LAAEI and ∼60% (6/10) success rate at the 6-month follow-up. The velocity of blood flow in the LAA typically decreases after LAAEI, and no blood clot formation in the LAA was detected during the follow-up period when patients were receiving routine OAC. The restoration of LAA flow velocity primarily depends on the passive systolic and diastolic movement of LAA and the possibility of reconnection. The utilization of ICE for transseptal puncture was both secure and effective, resulting in a notable reduction in radiation exposure duration. However, this conclusion is limited to animal study, and more clinical data are needed to further illustrate the safety and accessiblity of LAAEI in humans.

## Data Availability

The data that support the findings of this study are available from the corresponding author upon reasonable request.
